# Gamma Radiation-Induced Rib Necrosis and Stereotactic Radiosurgery Failure

**DOI:** 10.7759/cureus.14302

**Published:** 2021-04-05

**Authors:** James El Haddi, Christina R Layton, Ulugbek Negmadjanov, John Roberts

**Affiliations:** 1 Surgery, Florida Atlantic University, Boca Raton, USA; 2 Thoracic Surgery, Boca Raton Regional Hospital/Lynn Cancer Institute, Boca Raton, USA

**Keywords:** stereotatic radiosurgery, gamma radiation, rib osteonecrosis, radiation side effects

## Abstract

Stereotactic radiosurgery, or SRS, uses focused beams of gamma radiation targeted to specific areas of the body and has been used for multiple forms of non-small cell lung cancer. In this article, the authors describe two incidental cases of osteonecrosis in patients who had previously undergone stereotactic radiosurgery with recurrence of tumor. While this is a known side effect of traditional radiation therapy, it has not been described in the context of stereotactic radiosurgery. Further, these lesions were immediately deep to a rib, which may have shielded the lesion, and led to SRS failure. Osteonecrosis of the rib is a rare clinical entity but has been found to occur with glucocorticoid use, bisphosphonates, radiation therapy, and radiofrequency ablation. In the authors' review of the literature on SRS for lung cancer and intrathoracic pathology, rib osteonecrosis was not described and has not been mentioned as a possible side effect. Patients who have undergone thoracic stereotactic radiotherapy may develop side effects of traditional radiotherapy. We identified two patients who developed rib osteonecrosis though that has not been previously described as an adverse effect of stereotactic radiotherapy. The patients described in this case did not have any radiographic evidence of disease on imaging, suggesting that further research is warranted on the diagnosis and management of this rare disease entity.

## Introduction

Stereotactic radiosurgery, or SRS, uses focused beams of gamma radiation targeted to specific areas of the body [1] and has been used for multiple forms of non-small cell lung cancer in addition to intrathoracic metastatic disease. Proponents argue that it decreases the toxic effects of radiation while maintaining therapeutic treatment of malignant lesions of primary and metastatic lung cancers [2-4]. Osteonecrosis of the rib is a rare clinical entity but has been found to occur with glucocorticoid use, bisphosphonates, radiation therapy, radiofrequency ablation and stereotactic radiosurgery [5-7]. In this article, the authors describe two incidental cases of rib osteonecrosis in patients who had previously undergone stereotactic radiosurgery with recurrence of tumor. Rib fractures and osteoradionecrosis are known consequences of stereotactic radiosurgery [8]. Here we describe the incidental discovery of rib fractures not detected on preoperative cross-sectional imaging. These cases suggest that further research is warranted on the diagnosis and management of this rare disease entity.

## Case presentation

Case 1

Presentation 

**Figure 1:**
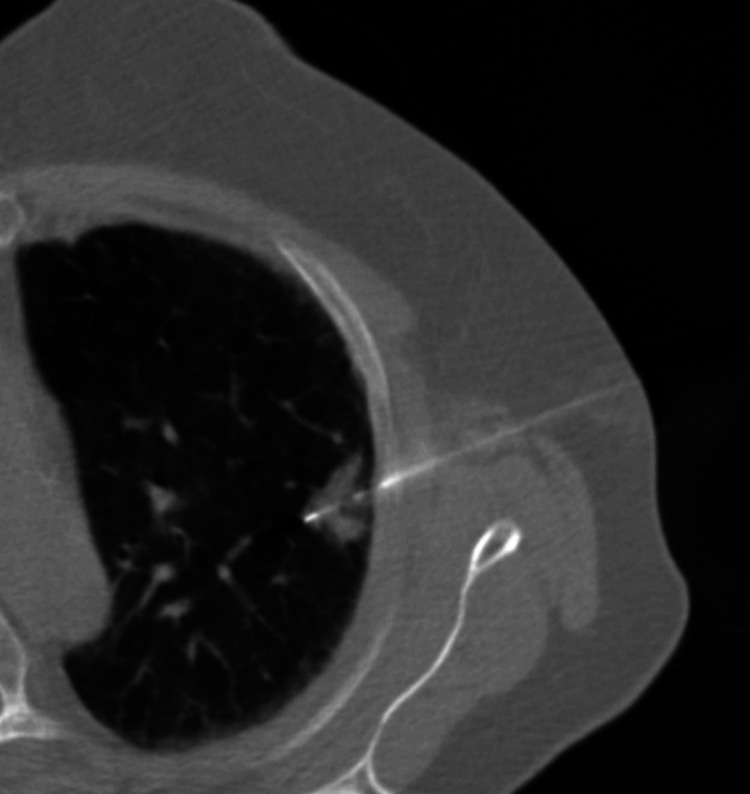
CT-guided biopsy of left upper lobe nodule CT: computed tomography

The first case involves a 78-year-old female with IV bronchogenic adenocarcinoma with involvement of the left upper, right middle, and right lower lobes. She had undergone previous chemotherapy and wedge resection with lymphadenectomy of the right middle and right lower lobe tumors. The patient also had undergone SRS of the left upper lobe two years prior to presentation. Treatments had been done both at our facility and at an outside institution. Despite these therapies, she was noted to have disease progression around the site of previous stereotactic therapy. After a discussion of the patient’s case at the multidisciplinary tumor board, the decision was made to proceed with resection of the left upper lobe. Preoperatively the patient had undergone CT-guided biopsy to confirm recurrence as shown in Figure 1.

Intervention 

**Figure 2:**
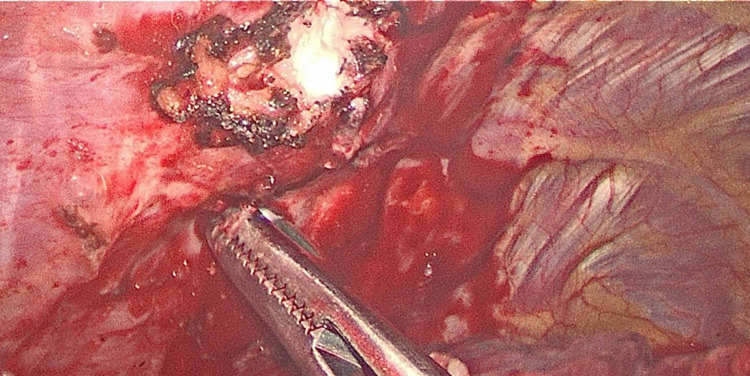
Intraoperative image of rib fracture

The patient was taken into the operating room and during the thoracoscopic evaluation of the left chest, there was noted extensive adhesion around the left upper lobe. After mobilizing and freeing the lung there was purulent drainage from the chest wall which was cultured and irrigated. Drainage was noted to be originating from a rib fracture and a fragment of the fractured third rib was resected and sent for pathological analysis. Intraoperative image is shown in Figure 2. We then proceeded with wedge resection of the apical posterior segment and mediastinal lymphadenectomy. 

Hospital Course and Follow Up 

The patient was admitted to the step-down unit. She was maintained on cefazolin given the purulent materials encountered, however, cultures were negative. Pathology of the bone fragment revealed necrosis without evidence of malignancy. The wedge resection was consistent with well-differentiated mucinous adenocarcinoma without lymph node involvement and negative margins. Her chest tubes were removed on hospital day three and she was discharged to home on oxygen. The patient continues palliative Taxol and has been doing well as of her one-year follow-up.

Case 2

Presentation 

**Figure 3:**
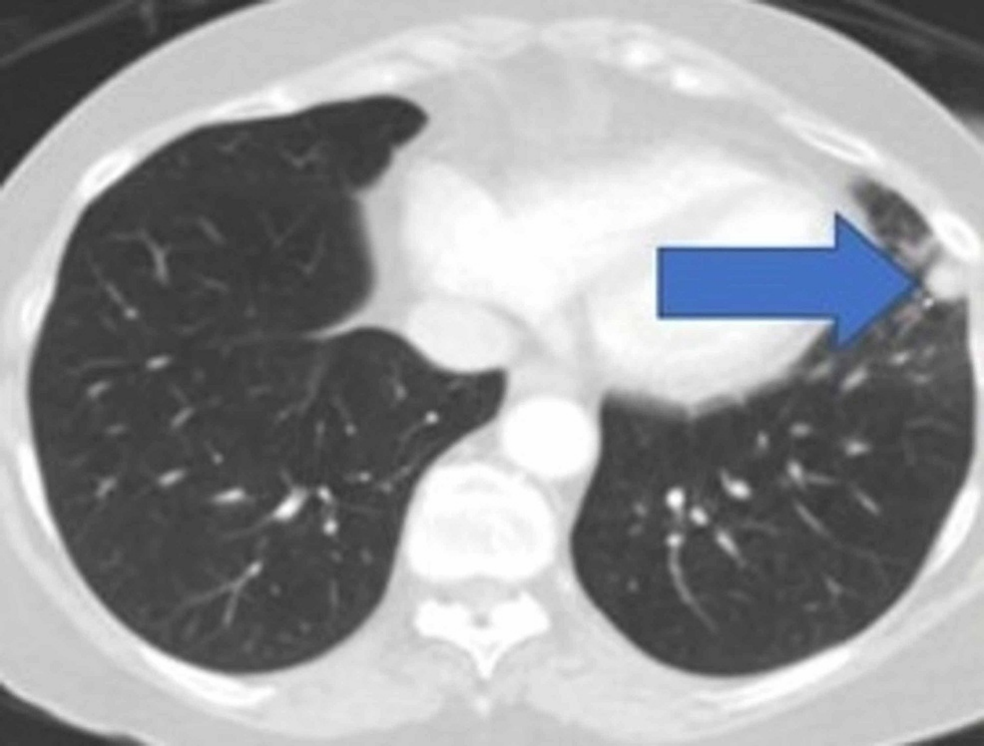
Chest CT. Arrow identifying left lower lobe nodule CT: computed tomography

A 60-year-old female with a history of gastrointestinal malignancy had previously undergone a pancreaticoduoduodenectomy after 12 cycles of FOLFOX therapy. Follow-up PET-CT was concerning for a metastatic left pulmonary nodule. This was further evaluated with a chest CT as shown in Figure 3. She was started on FOLFIRI and SRS of both lung nodules. Despite these therapies, there was progression of the nodule in the left lower lobe of the lung. Additionally, there was new thickening of the distal transverse colon. After discussion at the multidisciplinary tumor board one year after her completion of SRS the decision was made to proceed with staged metastectomy of the abdominal and left pulmonary lesions. The patient noted several months of left-sided chest-wall pain treated with lidocaine patches and no identifiable musculoskeletal abnormalities on preoperative imaging. Seven days after undergoing open left colectomy with lymphadenectomy, she did proceed to the operating room with the thoracic surgery team.

Intervention 

The patient was taken to the operating room and placed in the right decubitus position. Thoracoscopic evaluation was begun and there was bulging of the pleura noted adjacent to the lung lesion. Upon incision of the pleura there was a rush of necrotic material with an incidentally noted rib fracture. This was debrided and sent for both cultures and pathology. Frozen sections were performed but negative for evidence of malignancy. The standard wedge resection was then completed with lymphadenectomy. A chest tube was left in place and the patient was taken to the critical care unit for postoperative monitoring.

Hospital Course and Follow Up

The patient was noted to have an air leak postoperatively. The preoperatively placed epidural was removed on postoperative day two due to inadequate analgesia. Final pathology revealed adenocarcinoma, bowel primary, with no evidence of involvement of the lymph nodes or resected rib portion. On postoperative day three she was noted to have feculent drainage from her abdominal incision prompting laparotomy with right colectomy and ileosigmoidostomy for bowel ischemia and associated perforation. The patient had a prolonged recovery on broad-spectrum antibiotics and total parenteral nutrition (TPN) until bowel function was restored. The patient’s chest tube was transitioned to a one-way valve device for persistent air leak and discharged to home with home health care. Her chest tube was removed one month after discharge. She did develop an abdominal abscess which required percutaneous drain placement. Six months after her hospital course she was noted to have new mesenteric and hepatic lesions for which she underwent MRI-guided intensity-modulated radiation therapy. At one-year follow-up she had recovered well from her surgeries and subsequent abdominal radiation therapies and no longer had any left-sided chest wall pain.

## Discussion

Stereotactic radiosurgery, or SRS, has been utilized in early-stage inoperable non-small cell lung cancer patients since the early 2000s [9]. Since its adoption, attempts to expand the indications have been limited by recruitment [10]. Proponents have argued that there may be a role for expanding to elderly patients that may not otherwise tolerate surgical intervention [11]. Metastatic and recurrent disease is an ongoing topic of investigation [12]. As adoption of stereotactic radiosurgery expands in lung cancer, a greater understanding of the possible side-effects and indicators of failure is needed. Failure with SRS has been documented in patients with tumors larger than three centimeters and squamous pathology.

Radiation therapy, not exclusive to stereotactic radiation therapy, has a broad spectrum of side effects varying from minor skin hyperpigmentation to tissue necrosis [13]. While stereotactic therapy has been proposed to reduce toxic effects [14], dose-limiting pneumonitis and chest-wall pain has been reported [15]. Attempting to improve detection and define the safe limitations of these effects is ongoing [16-17]. 

Osteonecrosis of the rib is a rare clinical entity but has been found to occur with glucocorticoid use, bisphosphonates, radiation therapy, and radiofrequency ablation [5-7]. In the authors' review of the literature on SRS for lung cancer and intrathoracic pathology, rib osteonecrosis was not described and had not been mentioned as a possible side effect [18-19]. Animal models suggest that areas with high bone turnover such as the mandible and rib may both be increasingly susceptible to osteonecrosis when exposed to bisphosphonates [20] which suggests a similar susceptibility with radiation therapy. 

In general, osteonecrosis can be staged and diagnosed with imaging techniques such as bone scans, radiographs, and MRI [5-6]. Management ranges from surgical intervention to symptomatic palliation. In these patients, preoperative imaging including CTs and plain films did not reveal any evidence of osteonecrosis nor fracture. Had purulent drainage not been encountered intraoperatively, it is unclear what natural disease progression would have occurred. While these patients did not have any significant sequelae, the cases do demonstrate a rare consequence of SRS.

## Conclusions

Patients who have undergone thoracic stereotactic radiotherapy may develop side effects of traditional radiotherapy. Rib fractures and osteoradionecrosis are known consequences of stereotactic radiosurgery. Here we describe the incidental discovery of rib fractures not detected on preoperative cross-sectional imaging. These cases suggest that further research is warranted on the diagnosis and management of this rare disease entity.
